# Seizure prophylaxis in glioma surgery (SPRING): A multi-center, unblinded, randomized trial

**DOI:** 10.1093/nop/npaf114

**Published:** 2025-11-05

**Authors:** Michael D Jenkinson, Helen Bulbeck, Jackie Burns, Alasdair G Rooney, Richard Dobbie, Jade Carruthers, Sarah Lessels, Rachael Watson, Ciara Gribben, Tomos Robinson, Luke Vale, Gerard Thompson, Sara C Erridge, Colin Watts, Anthony G Marson, Robin Grant, For the SPRING study Collaborators

**Affiliations:** Institute of Systems, Molecular and Integrative Biology, University of Liverpool, Liverpool (M.D.J., A.G.M.); Department of Neurology, The Walton Centre NHS Foundation Trust, Liverpool (A.G.M.); Department of Neurosurgery, The Walton Centre NHS Foundation Trust, Liverpool (M.D.J.); brainstrust—the Brain Cancer People, Cowes, Isle of Wight (H.B.); Scottish Clinical Trials Research Unit, Public Health Scotland, Edinburgh (J.B., R.D., J.C., S.L., R.W., C.G.); brainstrust—the Brain Cancer People, Cowes, Isle of Wight (H.B.); brainstrust—the Brain Cancer People, Cowes, Isle of Wight (H.B.); brainstrust—the Brain Cancer People, Cowes, Isle of Wight (H.B.); brainstrust—the Brain Cancer People, Cowes, Isle of Wight (H.B.); brainstrust—the Brain Cancer People, Cowes, Isle of Wight (H.B.); Centre for Clinical Brain Sciences, University of Edinburgh, Edinburgh (A.G.R., G.T.); Centre for Clinical Brain Sciences, University of Edinburgh, Edinburgh (A.G.R., G.T.); Scottish Clinical Trials Research Unit, Public Health Scotland, Edinburgh (J.B., R.D., J.C., S.L., R.W., C.G.); Population Health Sciences Institute, Newcastle University, Newcastle upon Tyne (T.R., L.V.); Edinburgh Cancer Centre, NHS Lothian, Edinburgh (S.C.E.); Institute of Systems, Molecular and Integrative Biology, University of Liverpool, Liverpool (M.D.J., A.G.M.); Institute of Cancer and Genomic Science, University of Birmingham, Birmingham (C.W.); Department of Neurology NHS Lothian, Edinburgh (R.G.)

**Keywords:** clinical trial, glioma, levetiracetam, prophylaxis, seizure

## Abstract

**Background:**

50%-80% of glioma patients will have seizures. Guidelines recommend against prophylactic anti-seizure medication, but levetiracetam is frequently prescribed. Our aim was to determine the effectiveness of 12-month prophylactic levetiracetam at reducing seizure risk.

**Methods:**

Seizure-naïve patients undergoing glioma surgery were randomized (1:1) to 12-month levetiracetam or no prophylaxis and followed until death or a maximum of 18-months. The primary outcome was 1-year risk of first seizure. Patients who died within 12 months of randomization without experiencing a seizure were excluded from the primary outcome analysis. Target accrual was 804 participants. The trial was registered (ISRCTN 49474281 and EudraCT 2018-001312-30).

**Results:**

Between October 10, 2019 and August 30, 2022, 96 patients, from 24 to 79 years of age, were randomized to levetiracetam (*n* = 49) or no prophylaxis (*n* = 47). The trial closed early due to slow accrual and the Covid pandemic. In the levetiracetam group, 17 patients had a seizure and 32 did not (19 survived ≥12 months, 13 died within 12 months). In the no-prophylaxis group, 15 patients had a seizure and 30 did not (21 survived ≥12 months, 9 died within 12 months). Seventeen of the 36 evaluable levetiracetam patients (47%) had a seizure, compared with 15 of 36 evaluable no-prophylaxis patients (41%) (OR: 1.25, 95% CI, 0.49-3.21, *P* = .64). There were no levetiracetam-related serious adverse events.

**Conclusions:**

The seizure prophylaxis in glioma surgery (SPRING) trial provides no evidence of a difference between levetiracetam and no prophylaxis in the 12-month seizure risk in patients undergoing glioma surgery, but the study was underpowered. The role of prophylactic anti-seizure medication remains undefined.

Key Points

- There was no difference in 12-month seizure risk between those on levetiracetam and those with no prophylaxis.
- There were no serious adverse events related to prophylactic levetiracetam.

Importance of the study

This is the largest randomized trial for seizure prophylaxis in patients undergoing glioma surgery. Although the trial was underpowered and did not answer the research question, it nevertheless contributes to the existing literature base. Future systematic reviews can be updated and make use of individual patient data from SPRING for a meta-analysis. In the absence of any definitive evidence, national and international guidelines will continue to recommend that anti-seizure medication not to be used in the prophylactic setting for patients undergoing surgery for glioma, especially as the potential harms outweigh any benefits. Future clinical trials should be informed by the findings of SPRING, and an exploration of impacts of anti-seizure medications for this group of people using real-world data is needed.

Among patients who have glioma, 20%-25% will present with a new-onset seizure. Of the remaining 75%-80%, one-third to a half will develop seizures at some point during treatment or at a later stage prior to death.^1^^,^^2^^,^^3^ In the majority of cases, infiltrative gliomas are not curable, and consequently, optimizing symptom control is important for both patients and carers. Seizures have a major impact on quality of life and may result in injuries or life-threatening complications such as status epilepticus or aspiration pneumonia.^4^ Patients may also suffer debilitating anxiety about whether or when a seizure may occur.^4^

Anti-seizure medications (ASMs) are administered to those with seizures, but there remains controversy regarding the benefit of prophylactic ASM to seizure-naïve patients with glioma.^5^ The ability to prevent seizures is of great importance to patients and clinicians. There has been a shift away from the use of first-generation drugs such as phenytoin to newer generation ASMs such as levetiracetam.^6^ Guidelines from the Society for Neuro-Oncology (SNO) and the European Association for Neuro-Oncology (EANO) state that there is insufficient evidence to support prescribing prophylactic ASM at the point of diagnosis or the time of surgery, but these guidelines are based on trials in all brain tumors using older drugs such as phenytoin and valproate,^4^,^7^ meaning the evidence is both dated and not directly relevant to this clinical context. Despite this recommendation, prophylactic ASM continues to be prescribed preoperatively.^6^ Levetiracetam is the most frequently used ASM in patients with glioma since it is non-enzyme-inducing and therefore does not interact with dexamethasone, proton pump inhibitors, or chemotherapy. Levetiracetam has a lower side effect profile compared to older drugs but still causes fatigue and behavioral problems, which may be compounded by the symptoms from the glioma, surgery, and oncological ­treatment.^4^ Although short-course perioperative use is most common,^8^ there are observational studies suggesting that the duration of treatment prophylaxis is important, and those who receive prophylaxis for 6 months or more had significantly fewer seizures than those on prophylaxis for less than 1 month or not at all.^9^ This may be due to the higher risk of seizures at glioma relapse or prior to death. A systematic review recommended the future research should focus on the role of prophylactic ASM in the end-of-life phase, particularly in high-grade glioma patients whose survival is often measured in months,^10^ and that clinical trials should use newer ASMs such as levetiracetam.^5^^,^^7^

The Seizure Prophylaxis In Glioma multi-center randomized controlled trial (SPRING) was conducted to assess the clinical effectiveness of 12-months levetiracetam at reducing the risk of seizures, compared to no ASM, in seizure-naïve patients undergoing surgery for their glioma.

## Methods

### Study Design

In this multi-center, unblinded, randomized controlled trial, 12-months of prophylactic levetiracetam was compared to no ASM in seizure-naïve patients undergoing surgery for a newly diagnosed glioma. Trial sites were 14 regional adult neurosurgery and neuro-oncology centers in the United Kingdom and Eire (see Section 1 of the online supplementary Appendix). Ethics approval was obtained from the East of England—Essex Research Ethics Committee (ref: 18/EE/0389). The trial protocol is available at https://fundingawards.nihr.ac.uk/award/16/31/136 (substantial amendments are detailed in Section 6 of the online supplementary Appendix).

### Participants

To undergo randomization in the trial, patients had a cerebral glioma on MRI and planned for surgery (biopsy or resection), were ≥16 years old, and had a Karnofsky Performance Status (KPS) ≥70. Patients were excluded if they were pregnant, had a seizure in the 10 years prior to randomization, severe chronic kidney disease (CKD4— eGFR <30 ml/min), were taking concomitant methotrexate, other ASM or benzodiazepines, had hypersensitivity to levetiracetam, or had active suicidal ideation or severe depression as defined by a Patient Health Questionnaire-9 (PHQ-9) score of ≥20. Patients gave written informed consent.

### Randomization and Masking

Patients were randomized to 12 months of prophylactic levetiracetam or no ASM at a ratio of 1:1 using a minimization algorithm. The randomization sequence was stratified by neurosurgical unit, glioma grade (high vs low), and surgery type (biopsy or resection). Randomization occurred prior to surgery using the Edinburgh Clinical Trials Unit (ECTU) randomization system, a web-based randomization tool. Patients and clinicians were not blinded to the allocation or the assessment of outcomes.

### Procedures

Levetiracetam was taken orally in 2 daily doses of 500 mg, approximately 12 h apart. Patients with impaired renal function (CKD3, eGFR 30-59 ml/min/1.73 m^2^) started on a lower dose (25 omg bd) for the first 2 weeks and then titrated up (Table S1). All patients received a minimum of 2 doses prior to surgery and from 2 weeks to 12 months were taking 750 mg bd (or 500 mg bd in patients with impaired renal function). After completing 12 months of levetiracetam, patients who did not have a seizure were placed on a 6-week tapering regimen to discontinue the drug (Table S1).

Data were collected at pre-surgery (baseline), early postoperative assessment, and 3-monthly thereafter (Table S2). Patients were followed for a minimum 12 months and maximum 18 months. The types of data collected at each visit and the methods used are detailed in the online study protocol.

### Outcomes

The primary objective was to determine, in seizure-naive newly diagnosed cerebral glioma patients undergoing surgery, whether prophylactic levetiracetam preoperatively and for at least 1 year produces a meaningful (>50%) reduction in the risk of developing seizures when compared with standard care (no ASM). The corresponding primary outcome was the 12-month risk of first seizure. Seizures were defined as simple partial, complex partial, or partial seizure with secondary generalization (see panel). Seizures were assessed locally by the treating clinical team. In cases of diagnostic uncertainty, patients were reviewed by neurologists as part of the routine clinical pathway to confirm or refute the diagnosis. Excluded attacks were those deemed by the treating clinician not to be epileptic seizures.

### Panel: Seizure Definitions

1. Focal aware seizures (formerly “simple partial seizures”):
  - with motor symptoms: focal motor movements, versive/postural movements
  - with sensory symptoms: olfactory sensations
  - with autonomic signs
  - with psychic symptoms (eg, déjà vu, jamais vu)
2. Focal seizures with altered awareness (formerly “complex partial seizures”):
  - with impairment of consciousness only
  - with impairment of consciousness plus automatisms (lip smacking, fumbling, etc.)
3. Focal to bilateral tonic–clonic seizures (formerly “partial seizures with secondarily generalized seizures”):
  - Unconsciousness with generalized clonic movements
  - Unconsciousness with generalized tonic spasm, without clonic movements
  - Unconsciousness or staring with one of the following symptoms perceived by the patient:
    - A rising feeling from the abdomen to the throat
    - Smelling of odd scents
    - Stiffening or convulsions in the face or limb(s)
    - Turning the head to 1 side

Secondary outcomes were time to first seizure; time to first tonic–clonic seizure; patient-reported symptoms and adverse events; mood, personality, fatigue, and memory; progression-free survival (PFS); and overall survival (OS). Health economic outcomes were EQ-5D-5L quality-adjusted life years (QALYs) at 12-months and costs to the National Health Service (NHS) and personal social services. A safety analysis was performed only for those patients randomized to levetiracetam. Data on serious adverse events were collected (see online protocol).

### Statistical Analysis

A Trial Steering Committee, comprising a majority of independent members viewing reports blinded to treatment arm, and an Independent Data Monitoring Committee viewing unblinded reports reviewed the trial regularly to assess conduct, progress, including rates of seizures, and safety. The sample size for the primary outcome was calculated based on a 20% 12-month seizure rate in patients with suspected cerebral glioma after surgery. The trial hypothesis was a reduction in seizure rate to 10% in the treatment arm. Based on 90% power to identify an improvement in the 12-month seizure rate in the treatment arm compared to the control arm, 532 patients were required across the 2 arms, with a two-sided type I error level of 5%. Assuming a 24.8% 12-month mortality rate and that 12% of patients will be lost to follow-up, the final (maximum) sample size was 402 per arm.

The analysis was conducted according to a prespecified statistical analysis plan. Outcomes were analyzed according to the intention-to-treat principle with a 5% level of statistical significance and 95% CIs. The occurrence of seizures within 12 months of randomization was compared between study arms in those patients who survived for 12 months, using a logistic regression model fitted to the presence of a seizure within 12 months of randomization. The odds ratio (OR), associated 95% CIs, and two-sided *P*-values associated with the comparison of each arm are given. The estimated absolute difference in seizure rate is reported together with the associated 95% CI. The estimated absolute difference in seizure rate is reported together with the associated 95% CI.

For secondary outcomes, all patients were included, and the mean and median time to first seizure and time to first tonic–clonic seizure were calculated for each study arm, and the seizure-free probability within 12 months was analyzed using Kaplan–Meier plots. Progression-free survival and OS were also analyzed using Kaplan–Meier plots. The time-to-event distributions are statistically compared using log-rank tests based on the chi-square distribution with 1 degree of freedom. Due to the small number of events, the impact of covariates on time-to-event was not modeled using Cox Proportional Hazards. Quality of life, semantic verbal fluency test (SVLT), anterograde memory, and PHQ-9 scores were analyzed and presented for the 2 randomization groups, but no statistical comparisons were made due to the small patient numbers.

Primary outcome and safety analyses were validated by independent programming from the point of raw data extraction. All analyses were done using R packages in a Posit Workbench. The trial was registered with ISCTRN: 70051203 and EudraCT 2018-001312-30.

### Economic Analysis

The economic analysis adopted the perspective of the NHS and personal and social care services in the United Kingdom. As the SPRING trial was closed prior to reaching the recruitment target, the planned within-trial analysis and long-term economic model were considered no longer appropriate. Instead, health-related quality of life (HRQoL) and health service utilization data from the intention-to-treat population were described using summary statistics, but no formal statistical comparisons were made between the 2 arms.

The responses to the EQ-5D-5L were converted to utility scores using the mapping function developed by the NICE Decision Support Unit,^11^ using the “EEPRU dataset.”^12^ Data regarding the dispensing of the intervention drug for each participant in the intervention were calculated using information from the dosage and were micro-costed. The price of levetiracetam was gathered from the British National Formulary (BNF).^13^ Data on health service resource use of the trial participants in the 2 trial arms were collected using a health service utilization questionnaire administered to all participants at 3, 6, 9, and 12 months post baseline. Additionally, a Time and Travel questionnaire was completed at a single time point (6 months). Unit costs were gathered from the Unit Costs of Health and Social Care and the National Schedule of NHS Costs. The cost year of the analysis was 2023. The results from a standard gamble sub-study have already been reported.^14^

### Role of the Funding Source

The funders (NIHR and UCB Pharma) had no role in study design, data collection, data analysis, data interpretation, or writing of the report. The authors had full access to all the data in the study and share the responsibility for the decision to submit for publication.

## Results

**Figure 1.**
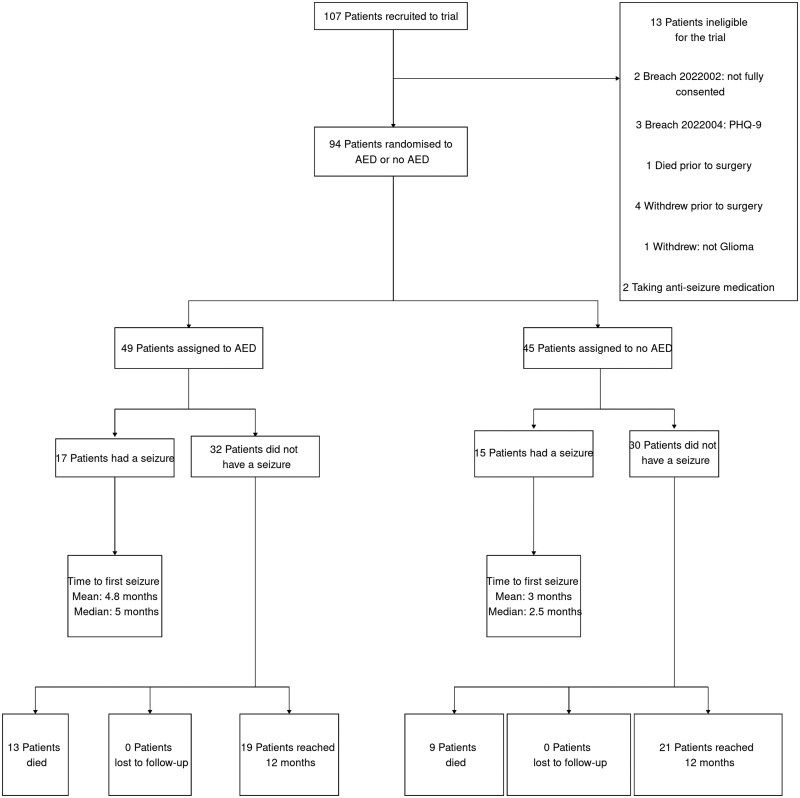
Trial profile. ASM, anti-seizure medication.

Between October 9, 2019 and August 31, 2022, 107 patients were recruited, of whom 13 were ineligible for randomization. There were 94 patients randomly assigned to the study arms (49 to levetiracetam and 45 to no prophylaxis; Figure 1). The characteristics of the 2 groups were similar at baseline (Table 1) and balanced for KPS (Table S3). All recruited patients had a glioma diagnosis, and the anatomical location, histopathology diagnosis, and molecular features are shown in Tables S4-S6.

**Table 1.** Baseline characteristics of the intention-to-treat population

	Intervention arm (levetiracetam)	Control arm (no prophylaxis)
**Age (years)**		
Median	61	59
Range	24-79	27-78
**Sex**		
Female	15	18
Male	34	27
**Glioma Grade (based on MRI)**		
High-grade glioma	43	41
Low-grade glioma	6	4
**Surgery**		
Biopsy	7	6
Resection	42	39

**Figure 2.**
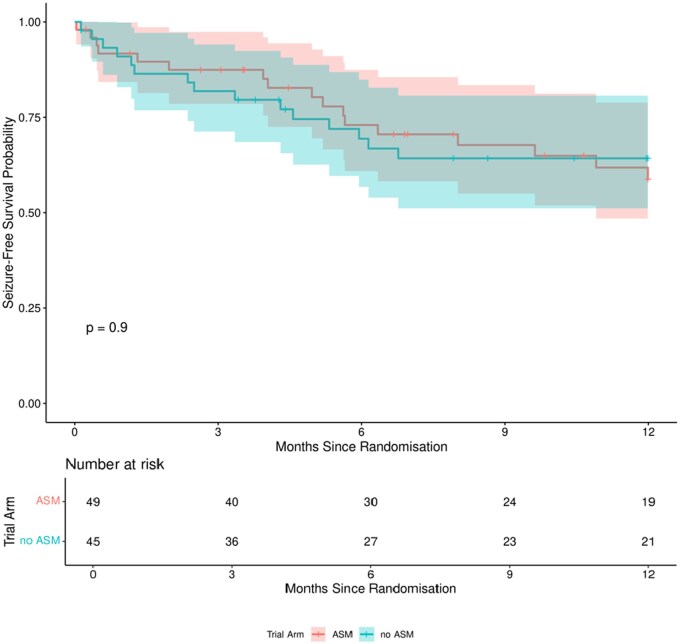
Kaplan–Meier curve showing time to first seizure for the whole study population. The numbers of patients at risk for each group is shown. The *P*-value for the log-rank test is greater than .05 (*P* = .9); therefore, the null hypothesis that there is no difference between groups in terms of the distribution of time until seizure can be rejected at the 5% level.

Patients who died within 12 months of randomization ­without experiencing a seizure (*n* = 13 in the levetiracetam group and *n* = 9 in the no-prophylaxis group) were excluded from the primary outcome analysis. Compared to the no-prophylaxis group (15 out of 36; 41%), the levetiracetam group (17 out of 36; 47%) had an increased likelihood of seizures (OR: 1.25, 95% CI, 0.49-3.21, *P* = 0.64), but the difference was not statistically significant. In patients who developed seizures, the median time to first seizure was 5 months in the levetiracetam group and 2.5 months in the no-prophylaxis group. For the whole study population, the 12-month seizure-free rate was 58.8% in the levetiracetam group and 63.8% in the no-prophylaxis group, and there was no statistically significant difference between the groups (Figure 2).

Thirteen patients had a tonic–clonic seizure, and the median time to first tonic–clonic seizure was 6.2 months in the levetiracetam group and 1.2 months in the no-prophylaxis group (Table 2). There were insufficient numbers to perform a statistical analysis between the 2 groups.

**Table 2.** Number of patients who had any type of seizure or a tonic–clinic seizure within 1-year of randomization according to trial arm

Trial arm	Patients	Any seizure (%)	Tonic–clonic seizure (%)	Median time to first tonic–clonic seizure (months)
Intervention arm (levetiracetam)	49	17 (34.7)	8 (16.3)	6.2
Control arm (no prophylaxis)	45	15 (37.5)	5 (11.1)	1.2
**Total**	**94**	**32 (34)**	**13 (13.8)**	**4.6**

Progression-free survival was not statistically different between the levetiracetam group (8.2 months) and the no-prophylaxis group (10.4 months) (Figure S1). The 12-month OS rate was 59.2% in the levetiracetam group and 68.9% in the no-prophylaxis group, and the difference was not statistically significant (Figure S2). In the levetiracetam group, 20 (41%) of 49 patients died within 12 months (median OS 6.8 months; range: 0.2-11.9), and in the no-prophylaxis group, 14 (31%) of 45 patients died within 12 months (median 4.6 months; range: 0.1-12.0).

The SVLT (the number of animals patients can name in 1 min) is a quick, repeatable method to measure executive function, language, and processing speed.^15^ At baseline, the median SVLT score was 18 (range: 3-40) in the levetiracetam arm and 18.5 (range: 2-30) in the no-prophylaxis arm (Table S7). Completion rates were 97.8% at baseline and progressively decreased during the trial to 40.4% at 12 months. The median scores ranged between 17 and 18 in the levetiracetam group and between 16 and 22 in the no-prophylaxis group (Table S7). Anterograde memory test scores showed a similar decline in completion rates, and scores were similar in both trial arms and remained static over 12 months (­Figure S3). The PHQ-9 is an instrument for screening and monitoring depressive symptoms. The percentage completion rates decreased at later points in the trial. Most patients reported PHQ-9 scores below 15, consistent with relatively mild depressive symptoms (Figure S4). Fatigue scores derived from the PHQ-9 Item 4 were similar in both groups at baseline, 3, 6, 9, and 12 months (Table S8). The Liverpool Impact of Epilepsy Scale^16^ and the Liverpool Seizure Severity Score^17^ measure the impact and severity of epilepsy on different aspects of a person’s life. Liverpool Impact of Epilepsy Scale scores ranged from 41 to 72 in the levetiracetam arm and 38 to 72 in the no-prophylaxis arm (Figure S5). Liverpool Seizure Severity Scores ranged from 34 to 44 in the levetiracetam arm and 35 to 49 in the no-prophylaxis arm (Figure S6).

The mean EQ-5D-5L utility scores by time point and intervention arm (Table S9) were similar across time points and intervention arm. At baseline and 3 months, the mean utility values were slightly higher in the no-prophylaxis group (0.807 and 0.768) as compared with the levetiracetam group (0.780 and 0.747). At 6 and 9 months, the mean utility values were slightly higher in the levetiracetam group (0.786 and 0.759) as compared with the no-prophylaxis group (0.748 and 0.723). At 12 months, the mean utility value in the no-prophylaxis group (0.804) was higher compared with the levetiracetam group (0.733). Mean QALYs by time point and intervention arm are shown in Table S10. The mean number of QALYs accrued is very similar between the no-prophylaxis group and the levetiracetam group at each time point.

The mean cost of the intervention per person was estimated to be between £231 and £254 per 3-month period (Table S11). At baseline, the mean total health care costs in the previous 3 months were very similar (£2580 in the levetiracetam group and £2532 in the no-prophylaxis group). At both the 3- and 6-month data collection points, the total health care costs were lower in the levetiracetam group (£1175 and £1278) compared to the no-prophylaxis group (£2703 and £2767). At the 9- and 12-month data collection points, the total mean health care costs were higher in the levetiracetam group (£1916 and £1238) compared to the no-prophylaxis group (£1597 and £686) (Table S12). Given the small sample sizes, no firm conclusions can be made from either the HRQoL or cost data.

In the patients taking levetiracetam, 7 reported severe depression and/or suicidal ideation, of whom 5 stopped the medication and withdrew from the trial, and 2 stopped the medication but stayed on trial. The median time to stopping levetiracetam was 196 days (range: 18-295 days). Two patients had a CTCAE grade 2 adverse reaction (somnolence requiring levetiracetam dose reduction and altered mood and depression leading to patient withdrawal). There were 13 Serious Adverse Events reported that were unrelated to the levetiracetam. There were no SUSARs (Table S13).

## Discussion

In this randomized controlled trial of ASM prophylaxis in seizure-naïve patients with glioma undergoing surgery, the 12-month seizure rate was 47% in those receiving 12-month prophylactic levetiracetam and 41% in those not receiving ASM. The study closed early due to slow accrual as a consequence of the COVID pandemic, with only 94 of the planned 804 patients recruited, and was underpowered to answer the primary research question. The study hypothesis was that prophylactic levetiracetam would increase the time to first seizure and reduce the severity of the first seizure should it occur. Time to first seizure was longer in the levetiracetam arm (5.0 months) compared to the no-prophylaxis arm (2.5 months), and time to first tonic–clonic seizure (a measure of seizure severity) was longer in the levetiracetam group (6.0 months) compared to the no-prophylaxis arm (1.2 months). Whilst these findings suggest a direction of effect favoring the use of prophylactic levetiracetam, the difference was not statistically significant and as such does not support a change in routine clinical practice.

The SPRING trial is the largest randomized study to date on this question and provides additional data to inform the debate on the use of seizure prophylaxis in glioma surgery, although it does not fundamentally alter the current knowledge base. Current EANO/SNO clinical practice guidelines do not recommend the use of seizure prophylaxis for patients with newly diagnosed brain tumors (primary and metastatic).^7^ These guidelines systematically reviewed the literature and rated the studies as class I (RCT), class II (cohort studies), or class III (case–control studies). There were 3 RCT (class I), 8 class II, and 11 class III studies suggesting that seizure prophylaxis is not effective.^17^ Only 1 class II study in melanoma metastasis^18^ and 1 class III study in glioblastoma^9^ supported their use. The recommendations are based mainly on lower-quality studies across a range of brain tumor types (primary and metastatic) and predominantly using first-generation ASMs such as phenytoin and valproate. The SPRING trial, although underpowered, adds further support to the recommendation not to use seizure prophylaxis.

Up to 1 in 4 patients with brain tumors have side effects from ASM that warrant a change or discontinuation.^19^ Compared to first-generation ASM, levetiracetam has a more favorable adverse effect profile^20^ but is still associated with fatigue (15%), behavioral problems (13%-38%), and aggression.^21^ All these symptoms can also arise from the glioma, surgery, radiotherapy, and chemotherapy. Whilst there is some evidence that levetiracetam may worsen these symptoms,^22^ other studies have suggested that levetiracetam may be neuroprotective in brain injury^23^ and that it may be associated with improved cognition in brain tumor patients.^24^ In the SPRING trial, there was no difference in mood, personality, fatigue, and memory at any time point between the 2 trial arms. These data should be interpreted with caution, due to the low number of patients and the inevitable attrition in data completeness at the 6-, 9-, and 12-month time points. It should be noted that 7 patients in the levetiracetam arm withdrew from the trial or stopped the medication due to severe depression or suicidal ideation. Consequently, in addition to there being no difference in seizure rates, time to first seizure, and seizure severity, levetiracetam may be causing harm with no benefit.

Levetiracetam may have an oncological benefit for patients taking the alkylating chemotherapy drug temozolomide.^25^ Poor-quality retrospective studies, with significant risk of bias, have reported increased progression-free and OS in patients with glioblastoma who received levetiracetam prophylactically or for seizures compared to those that did not^26-28^ although data from a systematic review do not support this finding.^7^ In the SPRING trial, there was no difference in progression-free or OS between the 2 treatment arms. A trial to determine the efficacy of levetiracetam on survival in glioblastoma would likely face similar recruitment challenges.^26^

Seizures have an impact on quality of life and cause uncertainty and anxiety for patients and carers.^4^ Seizures have an associated healthcare cost due to unscheduled admission to emergency departments. In the SPRING trial, there was a lower mean level of health care costs in the levetiracetam group compared with the no-prophylaxis group at 3 and 6 months; however, at 9 and 12 months, mean health care costs were higher in the levetiracetam group. Health-related quality of life was similar in both arms across all time points. None of the data on costs or HRQoL should be taken as any evidence of differences in HRQoL and cost. A definitive ­clinical trial combined with long-term economic modeling is still needed to fully understand the economic implications of ASMs for patients undergoing glioma surgery.

The strengths of this study are that: (i) despite early ­closure, this was the largest randomized controlled trial to date to investigate the role of prophylactic ASM in patients undergoing glioma surgery; (ii) the study investigated prolonged seizure prophylaxis over 12 months; (iii) the study included both low- and high-grade glioma, balanced across each arm; (iv) participants were recruited across the whole of the United Kingdom to encompass adults of all ages and socio-economic classes, and there was no loss to follow-up; (v) the completion rates for the health economics measures (EQ-5D-5L and heath care utilization questionnaire) were good, implying that the measures were fit for purpose and could be used in a future, definitive study.

**Figure 3.**
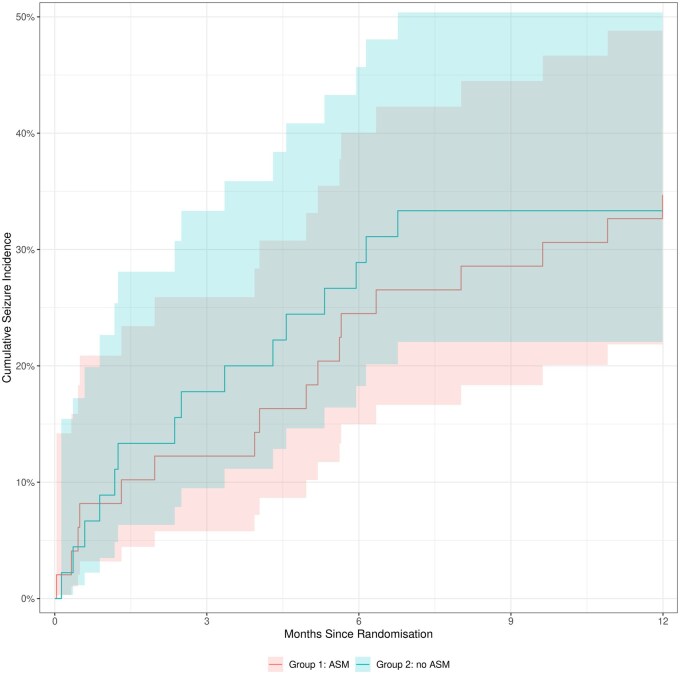
Cumulative incidence of seizures over 12 months for the whole study population. The 12-month seizure risk was 34.7% in the ASM group (95% CI, 21.6%-48.1%) and 33.3% in the no ASM group (95% CI, 20.0%-47.2%) (*P* > .5; Gray’s test).

Some limitations of the trial should be noted. First, it was not possible to blind participants and researchers, as this was an open-label trial. This was an intentional part of the design since it was considered more pragmatic to deliver, and pre-trial work with patients, carers, and charities showed that administering a placebo in patients with a limited life expectancy was not acceptable, and that the primary outcome measure was less susceptible to a placebo effect. Second, approximately one-quarter of the participants died from their glioma within 12 months and therefore did not reach the primary outcome of seizure rate at 12 months. To address this limitation, a post-hoc analysis with death as a competing event showed that there was no statistically significant difference between the 2 arms in the cumulative risk of developing seizures at 12 months (Figure 3).^29^ Finally, the study was underpowered and closed early due to slow recruitment impacted by the COVID-19 pandemic, and we cannot fully exclude a difference between the 2 arms that could favor prophylaxis.

In conclusion, the SPRING trial is the largest prospective randomized study of seizure prophylaxis in glioma surgery worldwide. The study closed early and suffered from poor accrual caused by the COVID-19 pandemic. Although the study is underpowered, based on the limited data, there was no difference in the 12-month seizure risk in the prophylaxis and no-prophylaxis arms. The wide CIs mean that we cannot be certain that a difference exists. The study provides the highest quality data available that could be combined with other studies for a future individual patient data meta-analysis. Current national and international guidelines will continue to advise against the use of seizure prophylaxis. A definitive clinical trial is still needed to answer this important clinical question.

## Supplementary Material

npaf114_Supplementary_Data

## Data Availability

Individual participant data that underlie the results reported in this article, after deidentification, will be made available. The study protocol, statistical analysis plan, and consent forms will also be made available. Data will be available beginning 9 months and ending 3 years after publication. Data will be available to researchers whose proposed use of the data is approved by the original study investigators. Proposals should be directed to the corresponding authors and requestors will need to sign a data access agreement.

## Lay Summary

Seizures affect up to 1 in 4 people with glioma, a type of brain cancer. We do not know if taking anti-seizure medicine (such as levetiracetam) before a seizure starts can help. The SPRING trial tested whether taking levetiracetam for 12 months after glioma surgery could prevent seizures in people who had never had one. Ninety-six people took part and were randomly assigned to receive levetiracetam or not. After 1 year, both groups had a ­similar number of seizures. No serious side effects were found. Although the study was small, it helps us understand more about seizure prevention in people with glioma.
